# Reconstruction of a Global Transcriptional Regulatory Network for Control of Lipid Metabolism in Yeast by Using Chromatin Immunoprecipitation with Lambda Exonuclease Digestion

**DOI:** 10.1128/mSystems.00215-17

**Published:** 2018-07-31

**Authors:** David Bergenholm, Guodong Liu, Petter Holland, Jens Nielsen

**Affiliations:** a Department of Biology and Biological Engineering, Novo Nordisk Foundation Center for Biosustainability, Chalmers University of Technology, Gothenburg, Sweden; b Novo Nordisk Foundation Center for Biosustainability, Technical University of Denmark, Hørsholm, Denmark; Luxembourg Centre for Systems Biomedicine

**Keywords:** ChIP-exo, transcriptional regulatory network, environmental response, lipid metabolic map, novel targets

## Abstract

To build transcription regulatory networks, transcription factor binding must be analyzed in cells grown under different conditions because their responses and targets differ depending on environmental conditions. We performed whole-genome analysis of the DNA binding of five Saccharomyces cerevisiae transcription factors involved in lipid metabolism, Ino2, Ino4, Hap1, Oaf1, and Pip2, in response to four different environmental conditions in chemostat cultures, which allowed us to keep the specific growth rate constant. Chromatin immunoprecipitation with lambda exonuclease digestion (ChIP-exo) enabled the detection of binding events at a high resolution. We discovered a large number of unidentified targets and thus expanded functions for each transcription factor (e.g., glutamate biosynthesis as a target of Oaf1 and Pip2). Moreover, condition-dependent binding of transcription factors in response to cell metabolic state (e.g., differential binding of Ino2 between fermentative and respiratory metabolic conditions) was clearly suggested. Combining the new binding data with previously published data from transcription factor deletion studies revealed the high complexity of the transcriptional regulatory network for lipid metabolism in yeast, which involves the combinatorial and complementary regulation by multiple transcription factors. We anticipate that our work will provide insights into transcription factor binding dynamics that will prove useful for the understanding of transcription regulatory networks.

**IMPORTANCE** Transcription factors play a crucial role in the regulation of gene expression and adaptation to different environments. To better understand the underlying roles of these adaptations, we performed experiments that give us high-resolution binding of transcription factors to their targets. We investigated five transcription factors involved in lipid metabolism in yeast, and we discovered multiple novel targets and condition-specific responses that allow us to draw a better regulatory map of the lipid metabolism.

## Abstract (precis)

Transcription factors play a crucial role in the regulation of gene expression and adaptation to different environments. To better understand the underlying roles of these adaptations, we performed experiments that give us high-resolution binding of transcription factors to their targets. We investigated five transcription factors involved in lipid metabolism in yeast, and we discovered multiple novel targets and condition-specific responses that allow us to draw a better regulatory map of the lipid metabolism.

## INTRODUCTION

Cellular functions are reprogrammed in response to environmental changes, and transcription factors (TFs) play a key role in this regulation. The complexity of transcriptional regulation in eukaryal cells and the lack of knowledge regarding the structure of regulatory networks, however, limit understanding of how this type of reprogramming occurs. Approximately 200 sequence-specific TFs have been characterized or predicted in the model organism Saccharomyces cerevisiae (1). In transcriptional regulatory systems of low connectivity (such as that in Escherichia coli [2–4]) or in systems that have hierarchical structures, TF deletion studies have proved to be useful, as the resulting phenotype corresponds well with the function of the deleted TF. However, in S. cerevisiae where a hierarchical structure of transcription factors does not exist due to the complex regulation with internal loops where TFs are controlling each other (5) and where genes are regulated by multiple TFs, deletion of individual TFs followed by genome-wide transcription analysis has not allowed us to identify the full function of TFs (6). To better resolve these combinatorial regulations and internal regulatory loops, it is necessary to identify the binding sites of transcription factors at a high resolution and their changes in response to environmental conditions. Here we demonstrate that by using this approach, it is possible to reconstruct a transcriptional regulatory network for lipid metabolism in yeast by mapping binding of five TFs that are involved in regulation of lipid metabolism: Ino2, Ino4, Hap1, Oaf1, and Pip2.

To generate a genome-wide lipid metabolic transcriptional regulatory network, we used chromatin immunoprecipitation with lambda exonuclease digestion (ChIP-exo) (7), followed by high-throughput sequencing, to map binding of these five TFs. Earlier studies on the genome-wide binding of these TFs were done mainly in rich media using chromatin immunoprecipitation with microarray technology (ChIP-chip) (8), which does not allow the precise binding sites to be mapped or evaluation of conditional binding under different conditions. The use of different culture conditions to map differential binding of TFs has been suggested (9), and recently, we showed that this allowed the identification of new functions of the transcription factor Cst6 (10).

Ino2, Ino4, Hap1, Oaf1, and Pip2 all play important roles in the lipid metabolic network of S. cerevisiae. Ino2 and Ino4 are paralogues that belong to the basic helix-loop-helix (bHLH) family and form a heterodimer that is involved in the phospholipid biosynthetic pathway (11). The YeTFaSCo database gives the consensus DNA-binding motif for the two TFs as CACATGC (12), which is also called the UAS_INO_ motif (13). Single deletions of either Ino2 or Ino4 yielded a decrease in phospholipid biosynthesis relative to the wild type, whereas the two regulators also have some different targets according to the transcriptome analysis (14). Hap1 is a Zn_2_Cys_6_ zinc finger TF that responds to oxygen and heme levels. Heme acts as an activator for Hap1 and the expression of the Rox1 repressor, and together, they form a circuit that fine-tunes the control of the oxygen-responsive pathways (15). The previously reported consensus motif for Hap1 is CCGXTAXXXCCG (16). Oaf1 and Pip2 are also C_6_ zinc finger TFs involved in β-oxidation and peroxisomal biogenesis, which can act as a heterodimer to bind oleate-responsive elements (OREs) (17), but they can also act independently of each other (18). The previous reported consensus motif for the Oaf1-Pip2 heterodimer is CGGXXXTX(7–10)CCG (19).

In our study we used chemostat cultures to generate chromatin binding data because this allows the analysis to be performed under well-controlled growth conditions and operation under different environmental conditions at the same specific growth rate. Four different limited conditions regarding different nutrition and oxygen availabilities were used to cover a wide range of different environmental growth conditions for S. cerevisiae.

## RESULTS

### Chemostat cultures and ChIP-exo mapping.

Strains expressing each of the five transcription factors (TFs) were constructed to have an *in situ* C-terminal tandem affinity purification (TAP) tag to enable immunoprecipitation of the TF. Each strain was cultured in chemostats with different limiting conditions in biological duplicates: nitrogen (N-lim), glucose (Glu-lim), glucose and oxygen (Ox,Glu-lim), and ethanol (Et-lim). Samples from each chemostat experiment were used to cross-link and purify DNA-protein complexes and digest nucleotides not covered by the cross-linked protein by the ChIP-exo method. Following release from the DNA-protein complex, the DNA was sequenced, and the reads were aligned to the reference genome assembly R64-2-1 of S. cerevisiae S288C. The identified TF-binding events were assigned to the closest gene and further analyzed for their biological impacts.

### ChIP-exo identified high-resolution targets of the five TFs.

**FIG 1:**
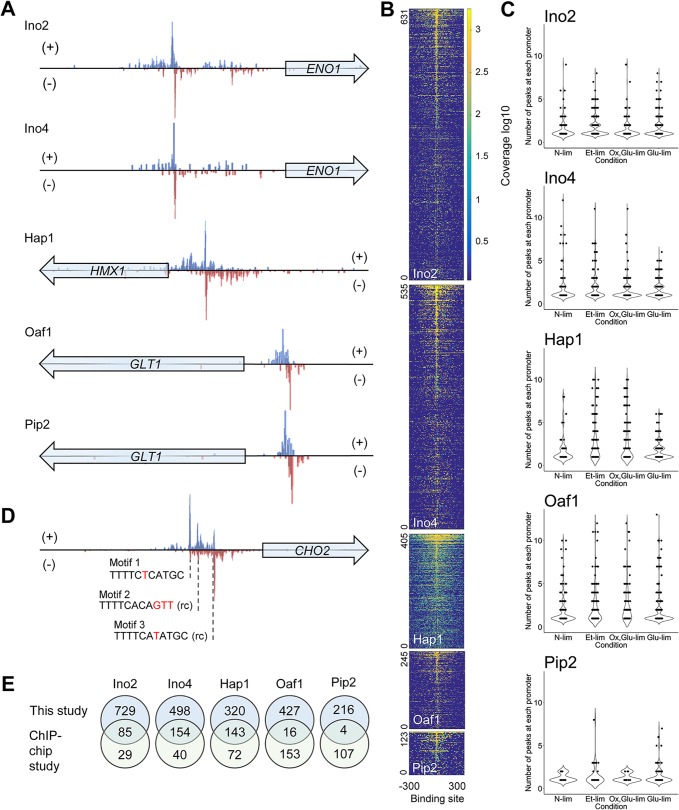
Identification of direct target genes of TFs by ChIP-exo. (A) Examples of TF-binding sites identified at high resolution. The distribution of 3′-trimmed sequencing reads mapped to forward (+) and reverse (−) strands of gene promoters are shown. (B) Heat map showing the binding of TFs on all target regions under the glucose-limited (Glu-lim) condition. Coverage values indicate the counts of reads around the center of binding sites. For each TF, the rows representing binding events are sorted by *S*/*N* ratio and aligned to their centers. (C) Violin plots of each TF and their peak count for each promoter. Most promoters have one or two binding sites per TF, but there can be even higher numbers. (D) Multiple binding sites of Ino2 on the promoter of *CHO2* under Et-lim condition. Three binding motifs of Ino2 are shown with sequence variations relative to the consensus motif (CACATGC) colored in red. rc, reverse complement sequence. (E) Comparison of the target genes encoding proteins under four conditions identified in this study and those in the previous ChIP-chip study with rich media (9). For the ChIP-chip data, genes with *P* value of <0.01 in the original data set are considered significant targets.

The binding events from the ChIP-exo experiments with each TF were analyzed with the peak-finding algorithm GEM (20). The identified events were then assigned a binding ratio, which corresponds to the signal-to-noise ratio (*S*/*N*), where the duplication showed a good correlation (see Fig. S1 in the supplemental material). ChIP-exo identified the binding positions of the TFs on target promoters with high resolution, which can be visualized for all five of the studied TFs (Fig. 1A). The reads from all events found were extracted 300 bp before and 300 bp after each event. The events were then aligned at the center to create a heat map of binding profiles, as shown in Fig. 1B. From these results, it is observed that the binding profiles of Ino2, Ino4, Oaf1, and Pip2 are narrow, whereas Hap1 has a broader binding profile. The broader binding of Hap1 seems to be a result of CCG/GCG-rich regions. Investigating the distribution of binding events on the promoter regions showed that 60 to 80% of all binding occurs within the 600 bp upstream of the ATG codon (Fig. S2). By investigating the promoter regions of identified genes, we found that many promoters have multiple binding sites for one TF (Fig. 1C). An example of such multiple binding is demonstrated in Fig. 1D, where Ino2 bound to three sites in the promoter of *CHO2*. The binding sequences for Ino2 in the *CHO2* promoter are not directional, and none of the identified binding sites have the exact above-mentioned Ino2 consensus motif.

10.1128/mSystems.00215-17.1 FIG S1  Correlation between duplicates of ChIP-exo. The duplicates with log_2_ binding ratios (*S*/*N*) of >1 are plotted. Download FIG S1, TIF file, 3.1 MB. Copyright © 2018 Bergenholm et al. 2018 Bergenholm et al. This content is distributed under the terms of the Creative Commons Attribution 4.0 International license.

10.1128/mSystems.00215-17.2 FIG S2  Distribution of peaks identified on the promoter regions. The promoter regions have been divided into 300-bp fragments where the number of peak summits have been summed. Download FIG S2, TIF file, 1.4 MB. Copyright © 2018 Bergenholm et al. 2018 Bergenholm et al. This content is distributed under the terms of the Creative Commons Attribution 4.0 International license.

We further compared all the identified targets for each TF (listed in Tables S1 to S5 in Data Set S1 in the supplemental material) to those identified in a previous ChIP-chip study where yeast was grown unlimited in rich media (9) (Fig. 1E). For Ino2 and Ino4, we confirmed most of the previously reported targets but also found hundreds of new targets. For Hap1, 143 targets overlapped with the targets of previous reports in the literature, although we missed 72 targets that had previously been reported, possibly due to the different culture conditions used or decreased significance of some peaks after data treatment (Fig. S3). For Oaf1 and Pip2, only a few targets overlapped with the previous ChIP-chip data. Oaf1 and Pip2 targets identified earlier by ChIP-chip are, however, questionable, as they did not identify most of the β-oxidation genes that are known Oaf1-Pip2-binding targets as evidenced by other studies (21) and our study (Table S8 in Data Set S1). Of the 85 oleate-responsive elements (OREs) previously predicted in putative promoter regions (18), 38 were identified to be bound targets of Oaf1 or Pip2. Combining the binding sites detected by us and the 15 ones identified previously (22), we expand the bound ORE targets for Oaf1 or Pip2 to 45.

10.1128/mSystems.00215-17.3 FIG S3  Effect of data treatment on the identification of Hap1-binding events. The binding on *ERG27* promoter is taken as an example, where the distributions of untrimmed 75-base reads (raw data) and 3′-trimmed reads (treated data) mapped to this region are shown. The binding of Hap1 can be manually identified from raw data (consensus motifs marked by yellow triangles) but was not detected using the treated data. This can lead to missing targets, which is a weakness in current high-throughput genome-scale analysis. Download FIG S3, TIF file, 0.5 MB. Copyright © 2018 Bergenholm et al. 2018 Bergenholm et al. This content is distributed under the terms of the Creative Commons Attribution 4.0 International license.

10.1128/mSystems.00215-17.10 DATA SET S1  Table S1 shows Ino2 data, including the peak loci, highest peaks [log]_2_(S/N)], motif, and direction and distance to ATG for each gene bound under each condition. Table S2 shows Ino4 data, including the peak loci, highest peaks [log]_2_(S/N)], motif, and direction and distance to ATG for each gene bound under each condition. Table S3 shows Hap1 data, including the peak loci, highest peaks [log]_2_(S/N)], motif, and direction and distance to ATG for each gene bound under each condition. Table S4 shows Oaf1 data, including the peak loci, highest peaks [log]_2_(S/N)], motif, and direction and distance to ATG for each gene bound under each condition. Table S5 shows Pip2 data, including the peak loci, highest peaks [log]_2_(S/N)], motif, and direction and distance to ATG for each gene bound under each condition. Table S6 shows primers used for strain construction and ChIP-qPCR. Table S7 shows adapters and primers used for ChIP-exo. Table S8 shows the overlap of binding targets of Oaf1 and Pip2 between this study and previous reports. Table S9 shows binding of TFs on lipid metabolism-related genes. Download DATA SET S1, XLSX file, 1 MB. Copyright © 2018 Bergenholm et al. 2018 Bergenholm et al. This content is distributed under the terms of the Creative Commons Attribution 4.0 International license.

**FIG 2:**
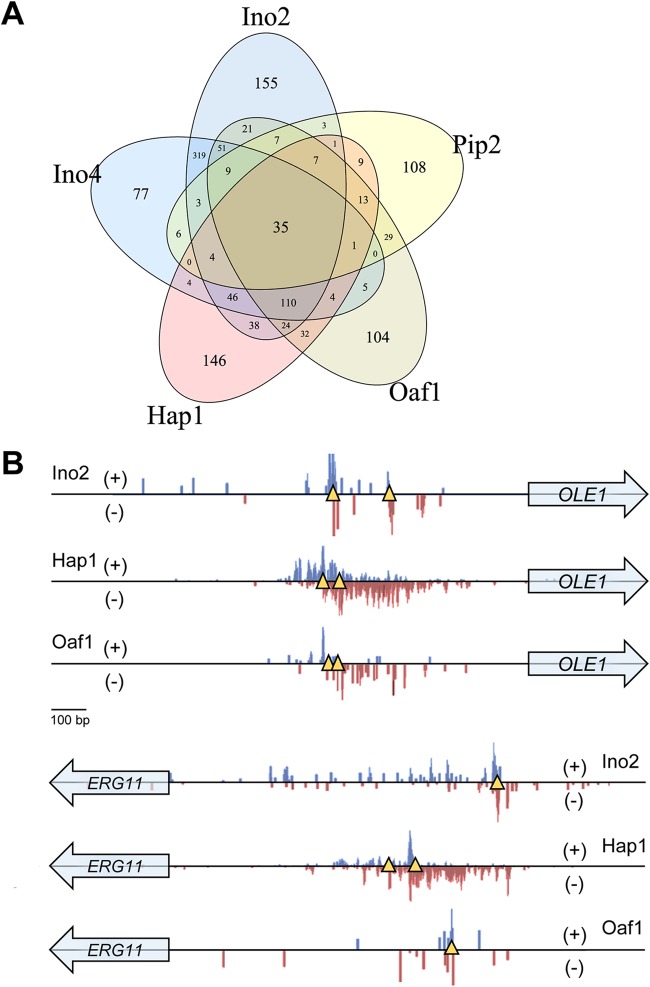
Combinatorial binding of TFs on target genes. (A) Venn diagram showing overlap of all the target genes of the five TFs. (B) Binding of Ino2, Hap1, and Oaf1 on the promoters of *OLE1* and *ERG11* under the Glu-lim condition. The distribution of 3′-trimmed sequencing reads mapped to forward (+) and reverse (−) strands of gene promoters are shown. Yellow triangles indicate putative binding sites of the corresponding TF according to the known consensus motif.

Because all the studied TFs are involved in lipid metabolism, some overlap between their targets was expected. Specifically, 35 genes were found to show binding of all five TFs (Fig. 2A), including seven genes involved in lipid metabolism. The overlapping targets for each condition are displayed in Fig. S4. Some of the shared targets are displayed in Fig. 2B, which shows the binding of three TFs on the promoter of *OLE1* encoding delta-9 fatty acid desaturase. The binding is highly concentrated in the same region. Looking closer at this region reveals that two binding motifs of Hap1 surrounded the binding sites of Oaf1 and Ino2, constituting a highly optimized region for responses to different signals, including oxygen levels, fatty acids, and membrane composition. This region overlaps with a previously reported region that has been called the fatty acid-responsive element (FAR) (23). With our data, we can now show which TF is responsible for the FAR. Figure 2B also displays the binding of three TFs on the promoter of *ERG11* encoding sterol 14-demethylase, where the binding sites are dispersed throughout the promoter region.

10.1128/mSystems.00215-17.4 FIG S4  Venn diagram showing the overlap of gene targets for TFs under each condition. Download FIG S4, TIF file, 1.3 MB. Copyright © 2018 Bergenholm et al. 2018 Bergenholm et al. This content is distributed under the terms of the Creative Commons Attribution 4.0 International license.

### New insights into the consensus binding motifs.

**FIG 3:**
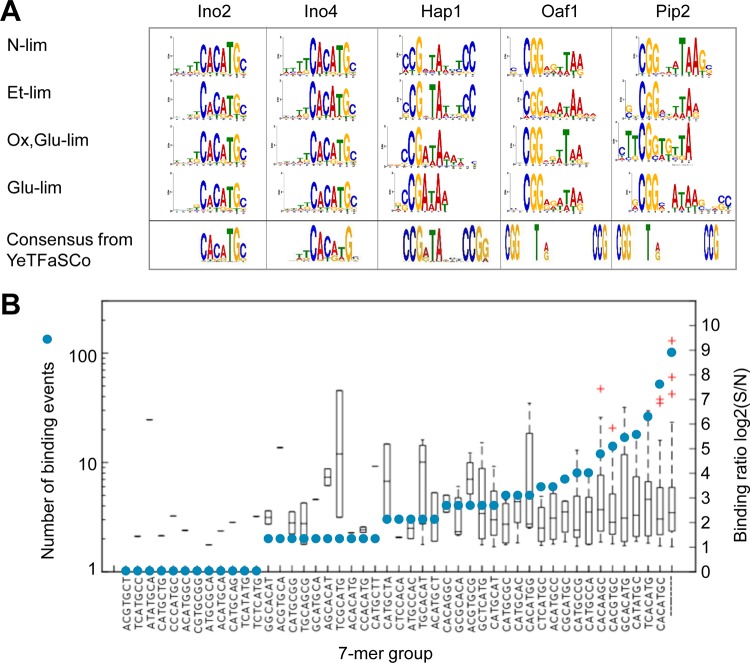
Consensus binding motifs of TFs. (A) Sequence logos of consensus motifs under different conditions. The motifs were identified using the MEME algorithm. Consensus motifs of the highest score for each TF in the YeTFaSCo database are shown for comparison. (B) Binding sequences of Ino2 under the Glu-lim condition. Blue circles show the numbers of binding events containing the indicated specific 7-mer sequence. Box plots show the binding levels of the events containing the sequence. For binding events containing multiple 7-mer sequences (e.g., TCACATGC containing both TCACATG and CACATGC), only the 7-mer with the most significant enrichment was counted.

We studied the motifs of binding sequences for each TF under each condition using the GEM software and then the MEME suite. The consensus motifs are displayed in Fig. 3A. For Ino2 and Ino4, the consensus motif found was CACATGC, which is the previously reported consensus motif in the YeTFaSCo database (12). The consensus motifs of Hap1 were found to be variations of the previously reported consensus motif CCGXTAXXXCCG. For N-lim and Et-lim, CCGXTATXTCC was found to be the consensus motif, and for Ox,Glu-lim and Glu-lim, the consensus motif was CCGATA. For Oaf1, CGGXXXTAA was found as a consensus motif under all conditions where the previously reported motif was CCGXXXTXA. For Pip2, the previously reported motif is the same as for Oaf1. Here it was found that Pip2 has CCGXXXTA as a consensus motif under all conditions but with more variation at the four to six positions.

We calculated the distribution of binding ratio (*S*/*N*) and the number of binding events identified for each k-mer group in motif discovery. As seen in Fig. 3B (data for Ino2 at Glu-lim), we could not identify a distinction between 7-mer groups in binding ratio. There was, however, a preference toward two of the 7-mers, TCACATG and CACATGC, which account for ~41% of all binding events found. For N-lim, Ox,Glu-lim, and Et-lim, the two most common 7-mers account for 74%, 38%, and 57% of all binding events, respectively. Similar trends were observed for all the TFs and their consensus motif under all conditions.

### Condition-dependent binding.

**FIG 4:**
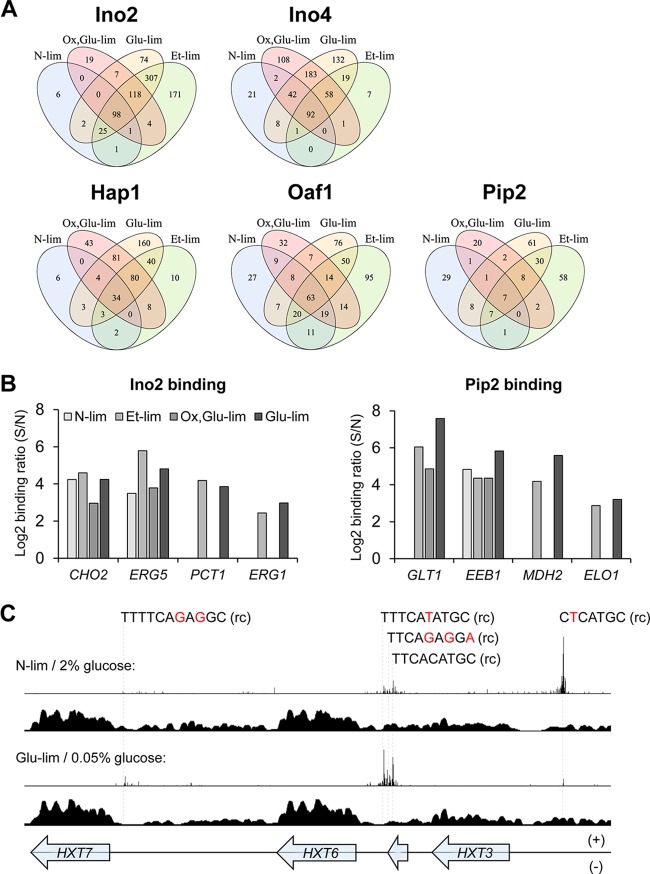
Growth condition-dependent binding of TFs. (A) Venn diagram of the TF targets and their overlap between conditions. Several genes for each TF are independent of the condition; however, many more genes are condition specific. (B) Differential binding of Ino2 and Pip2 on the promoter regions of specific target genes under different conditions. Binding ratios with log_2_ value below 1 are filtered out in data processing and are not shown. (C) Hexose transporter gene-rich region bound by Ino4 and nucleosomes under different conditions. For each group of conditions, the distributions of 3′-trimmed ChIP-exo reads of Ino4 (top plot) and sequencing reads of nucleosome-occupying regions (nucleosome-seq) (bottom plot [data from reference 24]) are shown. For nucleosome-seq data, cells were cultivated in synthetic complete medium containing 2% or 0.05% glucose, and DNA regions covered by sequencing reads indicate nucleosome-occupying regions. Sequence variations of binding sites relative to the consensus motif of Ino4 (CACATGC) are colored in red. rc, reverse complement sequence.

The different number of binding targets as well as altered occupancy levels in response to environmental changes were clearly observed for the five TFs (Tables S1 to S5 in Data Set S1). We investigated the overlap of gene targets between the conditions for each TF (Fig. 4A). Some gene targets are common for all conditions, which could be seen as a “core set” of gene targets. However, the interesting part is the shift of targets between conditions and thus their response to environmental changes. While constitutively binding to some targets (e.g., *CHO2* for Ino2 and *EEB1* for Pip2), Ino2 and Pip2 bind to additional targets (e.g., *PCT1* for Ino2 and *MDH2* for Pip2) only under respiratory metabolic conditions (Fig. 4B), resulting in higher number of targets under the latter conditions. This connection between metabolic states and TF binding indicates that the TFs have different biological functions by altering their targets in response to specific environmental or physiological signals.

One explanation for the differential binding could be different expression levels of the TFs under different conditions. However, the transcription level of each TF does not correlate with the number of targets identified for each condition (Fig. S5). This indicates that the range of targets is highly controlled by several factors such as posttranslational modifications of TFs and also chromatin structure (such as nucleosome occupancy) having an influence on the availability of targets for the TFs. The binding of Ino2 or Ino4 (Ino2/4) on a hexose transporter gene-enriched region provides some clues for the latter hypothesis. As shown in Fig. 4C, the promoters of hexose transporter genes *HXT3*, *HXT6*, and *HXT7* are occupied differently under N-lim and Glu-lim conditions by Ino4 (similar findings for Ino2 [data not shown]). We overlaid nucleosome distribution data adapted from reference 24, and even though the culture conditions are not completely the same in the two studies, the data integration makes sense from the aspect of glucose supply, and the results are striking. During growth at 2% glucose (glucose fermentation), the chromatin structure at the precise site of Ino2/4 binding is open at the *HXT3* site, whereas for *HXT6* and *HXT7*, the chromatin at the corresponding sites is closed. The opposite behavior can be observed during growth at 0.05% glucose (glucose respiration). This difference in chromatin structure is consistent with the different binding of Ino2/4 to this region between fermentative and respiratory metabolic conditions.

10.1128/mSystems.00215-17.5 FIG S5  Plotting of the transcription levels of the five TFs and the number of their binding genes under four growth conditions. No correlation could be observed between the two data sets. Download FIG S5, TIF file, 1.1 MB. Copyright © 2018 Bergenholm et al. 2018 Bergenholm et al. This content is distributed under the terms of the Creative Commons Attribution 4.0 International license.

### TF coordination of metabolic processes.

To investigate the different biological processes and functions potentially regulated by the TFs, we used genome-wide gene ontology (GO) sets. For each TF, we ran a gene set analysis using the Piano R package (25). For input, we used the log_2_(*S*/*N*) values and allowed GO terms that had *P* values of <0.01 to be selected as significant reporter terms (Fig. S6). While lipid metabolic process was confirmed as a significant target of all five TFs that we studied, many new functions were identified. For Ino2/4, we found that these TFs also bind genes in cell wall biogenesis and amino acid biosynthesis. The latter function was suggested in our previous study of *INO2-* or *INO4*-dependent genes through transcriptome analysis (14). Malate metabolism and glutamate biosynthesis, which are tightly connected to fatty acid utilization (26, 27), were identified as significant target processes of Oaf1 and Pip2.

10.1128/mSystems.00215-17.6 FIG S6  Reporter Gene Ontology process terms enriched by gene set analysis of TF-binding genes under different conditions. Target genes with a log_2_ binding ratio (*S*/*N*) of >1 were used for the analysis. A cutoff *P* value of <0.01 was applied to the terms. Download FIG S6, TIF file, 2.8 MB. Copyright © 2018 Bergenholm et al. 2018 Bergenholm et al. This content is distributed under the terms of the Creative Commons Attribution 4.0 International license.

Condition-dependent functions of the TFs were clearly revealed by the reporter GO term analysis of target genes. In line with the differential binding described above, Ino2 showed expanded binding under the respiratory metabolic conditions relative to the two fermentative metabolic conditions (Fig. S6). In addition to phospholipid biosynthesis as a constant target under all four conditions, Ino2 is associated with various processes, including cell wall biogenesis, methionine biosynthesis, and stress response under the respiratory metabolic conditions. Compared to Oaf1, the function of Pip2 targets is more affected by the culture conditions, with the highest number of reporter GO terms obtained under the Et-lim condition.

**FIG 5:**
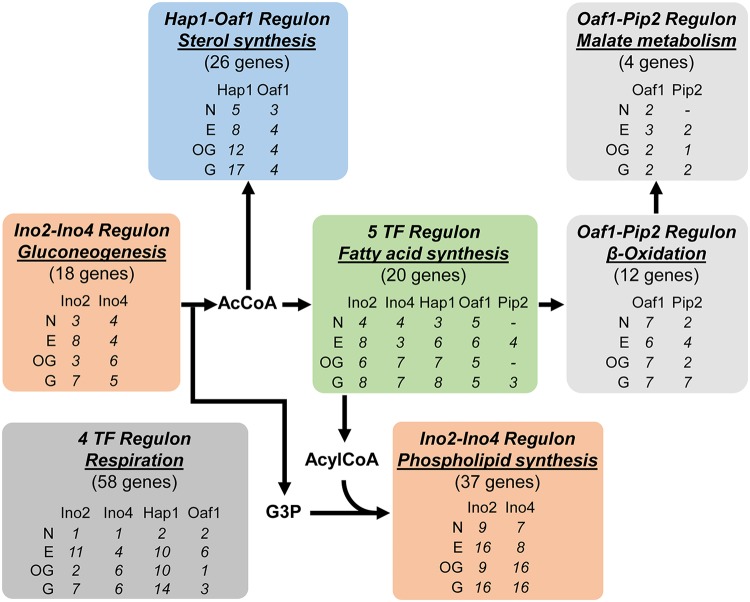
Coordination of lipid metabolism-related processes by TFs. The biological processes reported to have statistical significance (*P* value < 0.01) in gene set analysis of target genes with binding ratio log_2_(*S*/*N*) of >1 are shown. For a TF(s) significantly associated with a certain process, the number of bound genes is shown. A minus symbol indicates that the process is not significantly reported for the TF under the corresponding condition. Abbreviations of growth conditions: N, N-lim; E, Et-lim; OG, Ox,Glu-lim; G, Glu-lim.

The differential functions of TFs in regulating specific metabolic processes were further studied by investigating their targets with binding ratios higher than log_2_(*S*/*N*) of >1. Figure 5 summarizes the significant target processes related to lipid metabolism obtained for each TF under each condition, where each individual binding target can be found in Table S9 in Data Set S1. Ino2 and Ino4 bind genes encoding proteins involved in glycolysis and gluconeogenesis, pathways providing acetyl coenzyme A (AcCoA) for both fatty acid and sterol syntheses and glycerol-3-phosphate (G3P) for phospholipid synthesis, as well as their well-known binding to genes encoding proteins involved in phospholipid synthesis. Hap1 binds sterol synthesis genes which utilize AcCoA, with Oaf1 also being involved under respiratory metabolic conditions. Ino2, Ino4, Hap1, Oaf1, and Pip2 all bind to fatty acid synthesis genes, providing substrates for phospholipid synthesis, indicating the tight regulation of this process in response to various upstream signals. To recycle fatty acids, Oaf1 and Pip2 bind β-oxidation genes producing peroxisomal AcCoA as well as coordinated malate and glutamate metabolism. Hap1 also has the highest number of binding targets among the five TFs within respiration.

### Linking TF binding to transcriptional output and cellular lipid composition.

**FIG 6:**
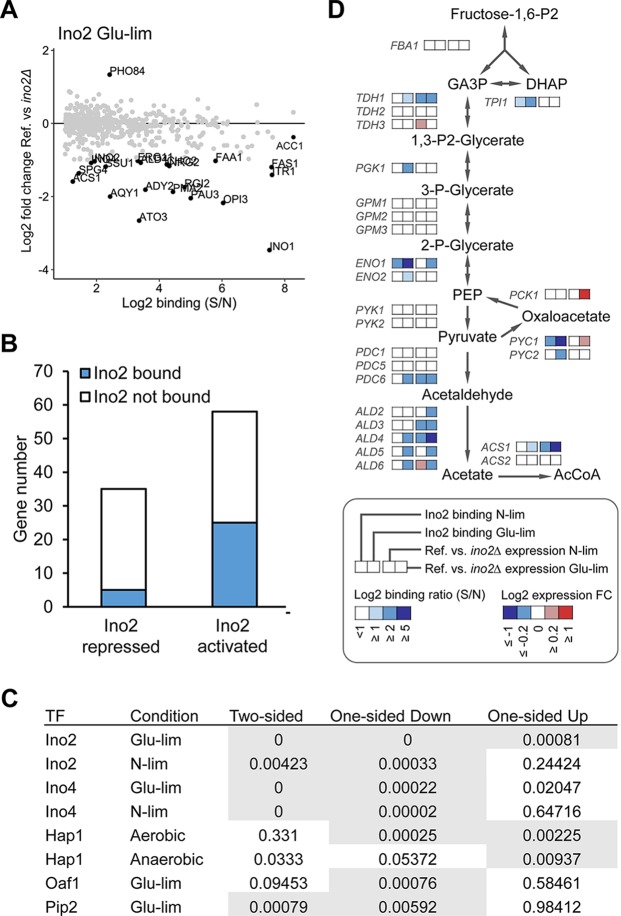
Data integration for elucidating the regulatory effect of TFs on their targets. (A) Effect of *INO2* deletion on the expression of Ino2-binding targets in Glu-lim chemostat culture. The binding ratio (*S*/*N*) of Ino2 determined by ChIP-exo (*x* axis) and the fold change (FC) in the level of expression in the *ino2*Δ deletion mutant relative to the level of expression in the reference strain determined by transcriptome analysis (*y* axis) are shown for each gene. Genes with a |log_2_(FC)| of >1 are highlighted. (B) Distinguishing direct targets from indirect targets within Ino2-dependent genes in Glu-lim chemostat culture. The Ino2-repressed and -activated targets indicate genes showing significant up- and downregulation (adjusted *P* value of <0.05), respectively, in the *ino2*Δ mutant strain relative to the reference strain. (C) *P* values from the *t* test of the five TFs under different conditions showed that in a one-sided *t* test, all TFs had significant (*P* < 0.01) down- or upregulation of genes that were bound compared to nonbound genes. Test results with a *P* value of <0.01 are shown on a gray background. References for the transcriptome data: Ino2 and Ino4 (14), Oaf1 and Pip2 (21), and Hap1 (31). (D) Identification of genes encoding components involved in central carbon metabolism as direct targets of Ino2. The binding ratio (*S*/*N*) of Ino2 and the fold change in the level of expression in the *ino2*Δ mutant strain relative to the level of expression in the reference strain in N-lim and Glu-lim chemostat cultures are shown. Abbreviations: Fructose-1,6-P2, Fructose-1,6-bisphosphate; GA3P, glyceraldehyde 3-phosphate; DHAP, dihydroxyacetone phosphate; PEP, phosphoenolpyruvate; AcCoA, acetyl-CoA.

The physical binding of a TF to a gene’s promoter does not necessarily mean that it regulates the transcription level, due to the dose- and condition-dependent feature of transcriptional regulation (28). Thus, integration of the direct target set of a TF identified by ChIP-exo with the targets transcriptionally affected by that TF is beneficial to understanding its biological function (29). We previously determined transcriptome changes in mutants lacking *INO2* or *INO4* relative to the wild type for similar growth conditions as applied here (14). When revisiting these data, we found the transcription of some targets strongly occupied by Ino2, e.g., *INO1*, *FAS1*, and *OPI3*, to be markedly impaired by the deletion of *INO2* (Fig. 6A). However, other strong targets, e.g., *ACC1*, are only slightly affected in the deletion mutant, suggesting dominant regulatory roles of other TFs on these promoters. The transcription of *PHO84*, which is another target of Ino2, showed a surprising increase in the *INO2* deletion mutant. This “repressing” effect is in agreement with the results of a previous study of an *INO2* overexpression mutant (30), which could be explained by a unique feature of the Ino2 binding sequence on the *PHO84* promoter determined here to be GCACGTGG, −415 to −408 bp relative to the start codon. This sequence has a mismatch of one nucleotide from the Ino2 consensus motif, but it exactly matches the consensus binding motif of another TF, Pho4 (12), which might trigger competition between the TFs.

By integrating the ChIP-exo and transcriptome data of TF deletion studies, we were able to distinguish direct and indirect targets of Ino2 within Ino2-dependent genes (Fig. 6B). For the lipid metabolic genes described above, we found most phospholipid synthetic genes to be direct targets of Ino2 (Fig. S7). The binding strengths are generally higher under the Glu-lim condition than under the N-lim condition, which may explain the stronger regulatory effects of Ino2 on targets under the Glu-lim condition (14).

10.1128/mSystems.00215-17.7 FIG S7  Regulation of lipid metabolic genes by Ino2. The binding ratio (*S*/*N*) of Ino2 and the fold change in expression level in *ino2*Δ relative to the reference strain in N-lim and Glu-lim chemostat cultures are shown. The major lipid components are shown in blue. Abbreviations: GA3P, glyceraldehyde 3-phosphate; DHAP, dihydroxyacetone phosphate; Ins, inositol; Etn, ethanolamine; Cho, choline; G3P, glycerol 3-phosphate; PA, phosphatidate; DAG, diacylglycerol; PI, phosphatidylinositol; PS, phosphatidylserine; PE, phosphatidylethanolamine; PC, phosphatidylcholine; PL, phospholipids; AcCoA, acetyl-CoA; HMG-CoA, 3-hydroxy-3-methylglutaryl-CoA; SE, sterol esters; TAG, triacylglycerol; FA, fatty acids. Download FIG S7, TIF file, 2.9 MB. Copyright © 2018 Bergenholm et al. 2018 Bergenholm et al. This content is distributed under the terms of the Creative Commons Attribution 4.0 International license.

As we could see few TF-bound genes that have been strongly affected by the deletion of a TF when using a strict cutoff of |log_2_(FC)| of >1 (FC stands for fold change) to define differentially expressed genes, we performed a *t* test to examine whether the bound genes for each TF had an altered expression or not. We used a two-sided *t* test (is there a difference between the bound genes and the nonbound genes in expression profiles), one-sided up *t* test (are the bound genes upregulated compared to the nonbound genes), and one-sided down *t* test (are the bound genes downregulated compared to the nonbound genes). We integrated TF deletion data for Ino2 and Ino4 from reference 14, Oaf1 and Pip2 from reference 21, and Hap1 from reference 31 (these studies used cultivation conditions similar to those used in this study). When a two-sided *t* test was used, some TFs showed significance. However, when splitting the TF deletion data into upregulated or downregulated genes in one-sided *t* tests, we found that for all TFs, the general trend was significant for downregulated genes and some showed significance for upregulated genes as well. The only exception was Hap1 under anaerobic conditions which was significant only for upregulated genes. This is consistent with the previous finding that Hap1 acts as a transcriptional repressor under anaerobic conditions (31). The resulting *t* test table can be seen in Fig. 6D, and the individual plots of FC and log_2_(*S*/*N*) can be seen in Fig. S8.

10.1128/mSystems.00215-17.8 FIG S8  Integration of TF deletion data and binding data. For each TF, the fold changes in the levels of gene expression in TF deletion mutants relative to wild type from published sources were incorporated with the binding data presented in this study. We highlight genes with a cutoff of |log_2_(FC)| of >1. Download FIG S8, TIF file, 1.3 MB. Copyright © 2018 Bergenholm et al. 2018 Bergenholm et al. This content is distributed under the terms of the Creative Commons Attribution 4.0 International license.

Ino2 and Ino4 are known to directly activate the transcription of several genes encoding components involved in central carbon metabolism, such as the enolase gene *ENO1* (32) and the AcCoA synthetase gene *ACS2* (33). Through data integration, we found more genes in central carbon metabolism as regulated targets of Ino2, including *TDH1*, *PDC6*, and three aldehyde dehydrogenase genes (Fig. 6C). Aldehyde dehydrogenases are responsible for converting acetaldehyde to acetate, which is on the primary pathway for AcCoA synthesis in S. cerevisiae (34). The coregulation of AcCoA and fatty acid biosynthetic genes highlights the importance of coordinating AcCoA supply with lipid biosynthesis (35).

To validate functional outcomes of our TF-binding data, we also constructed an *OAF1* deletion strain. Our data show that Oaf1 has a hierarchical role over some transcription factors, especially as it binds to the promoters of both Pip2 and Adr1 which also regulate fatty acid utilization. Another target of Oaf1 and Pip2 is *ELO1* which encodes the first elongase in fatty acid synthesis. If Oaf1 is controlling expression of *ELO1*, one would expect a decrease in long- and very-long-chain fatty acids, as *ELO1* elongates C_14_ to C_16_ species to C_18_ species (36). Fatty acids were extracted from the *oaf1*Δ strain, and we found that the strain does indeed have higher levels of shorter chains of fatty acids and low levels of longer-chain fatty acids (Fig. S8), which indicates that Oaf1 has a regulatory role over *ELO1*.

## DISCUSSION

Knowledge of the structure and function of the transcriptional regulatory network is important for understanding and engineering cellular processes (1, 37). Although considerable data have been accumulated on the regulatory interactions between TFs and their targets in S. cerevisiae (38), reconstruction of the transcriptional regulatory network is still challenging owing to the complexity of the network and the limitation of experimental techniques. Identifying direct binding sites is critical for elucidating the hierarchical structure of the regulatory network of TFs because doing so can distinguish direct and indirect targets. Here, we mapped the genome-wide binding targets of five TFs in S. cerevisiae using ChIP-exo, with a focus on the regulation of lipid metabolism. By using chemostat cultures, it was possible to examine the relationship between environmental conditions and TF behavior at a constant specific growth rate. As shown in Fig. 1, the advanced resolution of ChIP-exo compared with early ChIP methods enabled us to experimentally determine the distribution of multiple *cis*-acting elements across the genome. The result provides an *in vivo* picture of promoter structure, which could be the basis for precise engineering of promoters in response to specific conditions (39).

Previous studies of the five TFs investigated here mainly focused on their responses to specific environmental signals, such as inositol concentration, which affects the functions of Ino2 and Ino4 (40), and the presence of exogenous oleate, which activates Oaf1 (41). Under industrial cultivation conditions, S. cerevisiae is often exposed to changes in the species and concentration of carbon sources as well as dissolved oxygen levels. This motivated us to use four cultivation conditions representing these industrial growth conditions to compare the binding characteristics of the TFs. The results suggest that the binding of these TFs to the genome can be affected by factors other than their well-known specific signals. For example, the intracellular levels of phosphatidic acid, which acts as a central signal in the regulation of Ino2 and Ino4 activity (42), are similar between N-lim and Glu-lim conditions when high concentrations of inositol are present in the culture we used (43). However, the binding target set and regulatory effect of Ino2 are still quite different for the various conditions (see Fig. S7 in the supplemental material). Similar results were observed for Hap1, whose binding targets are different across the three aerobically growing conditions, although they have similar dissolved oxygen levels. We further found that the effect of metabolic status on TF binding could possibly be attributed to changes in chromatin accessibility resulting from the condition-dependent binding of other TFs, which can recruit chromatin remodelers (44). Exploring the causal relationship between chromatin remodeling and the binding of TFs would provide new understanding about the complex process of transcriptional regulation in eukaryotes.

The surprisingly high number of binding targets, together with the significant overlap of targets between the TFs, raises the question of whether all binding is functional. Through the integration of binding data and TF-dependent gene expression data, the contribution of TF binding to the corresponding gene’s transcription level was assessed. There is poor overlap between the binding targets and differentially expressed genes as classically defined, but by allowing all genes and all targets to be integrated in the analysis, we could find statistical significance for changes in expression. The data showed that the affected overall binding targets of the deleted TF were in fact downregulated and in one case upregulated. However, at the gene level, we could not see which targets would be most affected by said deletion or if they would be down- or upregulated. Looking at some individual TFs, we can see that for Ino2, there are more than 800 targets in our ChIP-exo data but only 20 genes were substantially affected in the deletion strain; it is therefore difficult to evaluate the regulation directly. Because Ino2 does efficiently regulate the expression of some targets (e.g., *INO1* under Glu-lim condition [Fig. 6A]), we believe the nonfunctional or less functional binding events observed under the same condition are due to not only the low transcriptional activation ability of Ino2 and Ino4 in the presence of exogenous inositol (45) but also the complex internal transcriptional regulatory loop and coregulation of genes by multiple TFs. The cumulative regulation mechanism has been supported by a previous study on *ENO1* expression, where a single gene deletion of the bound TFs each led to decreased but not abolished gene expression from the *ENO1* promoter (32). This is due to the fact that the remaining transcription factors can adapt to the deletion of one TF. Therefore, deletion of a TF may not result in the identification of true functional targets but rather the state of how well the cell is adapting. If, however, the TF is the sole activator/repressor of a gene or in a hierarchical position toward other TFs that are regulating the said gene, then the expression level of the gene might have a correlation with the function of the TF. These results highlight the importance of systematically mapping the binding sites of all TFs in the reconstruction of a genome-scale transcriptional regulatory network.

The emerging computer-assisted tools for pattern recognition using neural networks and deep learning algorithms may allow for better motif discovery (46–48) and help reveal the interplay of TFs on promoters. As we are starting to see highly dynamic and complex regulatory networks in eukaryal cells, such deep learning techniques could lead to the discovery and understanding of regulatory and condition-responsive elements rather than the hierarchical individual TF regulation and thus identify holistic transcriptional regulatory networks that are at the core of molecular biology.

## MATERIALS AND METHODS

### Strains.

The TF-tagging strains were constructed by transforming the uracil auxotrophic strain CEN.PK 113-5D of *Saccharomyces cerevisiae* (49) with a tagging cassette containing the tandem affinity purification (TAP) tag CBP-ProtA coding sequence and Kluyveromyces lactis *URA3* marker gene flanked by 45-bp sequences for homologous recombination at the TF gene locus (50). This integration allows the tag to be fused in-frame to the C-terminal end of each TF connected by a six-glycine linker. Transformants were screened on synthetic complete medium lacking uracil (SC_Ura; Formedium), and the correct integrations were identified by PCR. The function of the TAP tag was confirmed by ChIP followed by quantitative PCR (ChIP-qPCR) of known target promoters. All primers for cassette construction, PCR identification, and ChIP-qPCR are listed in Table S6 in Data Set S1 in the supplemental material. Transcriptome sequencing (RNA-seq) was performed on strain CEN.PK 113-5D complemented by *URA3* as well as a *HAP1*-TAP-tagged strain in all four chemostat conditions, where no significant changes could be identified between the two strains (unpublished data).

### Media and cultivations.

Single colonies from fresh agar plates were inoculated into 50 ml yeast extract-peptone-dextrose (YPD) medium in shaking flasks and grown for 12 to 24 h. Cells were harvested by centrifugation and resuspended in sterile water to obtain inoculum. Chemostat cultivation in liquid medium with a working volume of 500 ml was carried out using 1.2-liter DASGIP fermentors operated at 30°C with a dilution rate of 0.1 h^−1^. The pH was maintained at pH 5.0 using 2 M KOH. Minimal medium containing vitamin (1,000× stock solution [all amounts shown for 1 liter]; 0.05 g biotin, 0.2 g 4-aminobenzoic acid, 1 g nicotinic acid, 1 g calcium pantothenate, 1 g pyridoxine-HCl, 1 g thiamine-HCl, and 25 g *myo*-inositol) and trace metal (1,000× stock solution [all amounts shown for 1 liter]: 15.0 g EDTA-Na_2_, 4.5 g ZnSO_4_·7H_2_O, 0.84 g MnCl_2_·2H_2_O, 0.3 g CoCl_2_·6H_2_O, 0.3 g CuSO_4_·5H_2_O, 0.4 g Na_2_MoO_4_·2H_2_O, 4.5 g CaCl_2_·2H_2_O, 3 g FeSO_4_·7H_2_O, 1 g H_3_BO_3_, and 0.1 g KI) (51) as well as the following compounds (all amounts shown for 1 liter) were used for feeding: (i) for nitrogen-limited media, 1 g (NH_4_)_2_SO_4_, 5.3 g K_2_SO_4_, 3 g KH_2_PO_4_, 0.5 g MgSO_4_·7H_2_O, 60 g glucose; (ii) for glucose-limited media, 5 g (NH_4_)_2_SO_4_, 3 g KH_2_PO_4_, 0.5 g MgSO_4_·7H_2_O, and 7.5 g glucose; (iii) for oxygen- and glucose-limited media, 5 g (NH_4_)_2_SO_4_, 3 g KH_2_PO_4_, 0.5 g MgSO_4_·7H_2_O, 7.5 g glucose, 420 mg Tween 80, and 10 mg ergosterol; (iv) for ethanol-limited media, 5 g (NH_4_)_2_SO_4_, 3 g KH_2_PO_4_, 0.5 g MgSO_4_·7H_2_O, and 5 g ethanol.

Antifoam 204 at 0.05 ml liter^−1^ (aerobic cultures) or 0.2 ml liter^−1^ (Ox,Glu-lim culture) was added to the feeding media. For aerobic cultures, airflow of 30 liters h^−1^ and stirring speed of 600 rpm (Glu- and N-lim cultures) or 800 rpm (Et-lim culture) were used to keep the dissolved oxygen above 30% of air saturation. For Ox,Glu-lim culture, the fermentor was sparged with 30 liters h^−1^ of nitrogen gas with a stirring speed of 300 rpm. The oxygen and carbon dioxide in the off-gas were measured using the DASGIP GA4 exhaust analyzer after being cooled by a condenser operated at 4°C.

### ChIP-exo.

Cells cultivated in chemostats were sampled for ChIP-exo analysis after steady state was achieved (optical density at 600 nm [OD_600_], dissolved oxygen, and off-gas profiles became constant) for 48 to 60 h. Formaldehyde at a final concentration of 1% (wt/vol) and distilled water were added to the cultures to cross-link protein-DNA complexes at an OD_600_ of 1.0 and a total volume of 100 ml. Cross-linking was performed for 12 min at room temperature with shaking followed by quenching, washing, and freezing as previously described (10). ChIP-exo was performed according to a previously reported method (52) with modifications (10, 29). The first adapters contain unique 6-bp index sequences. The final DNA samples were pooled in equimolar amounts and sequenced on the NextSeq 500 system (2 × 75 bp, mid-output mode; Illumina). The ChIP-exo experiments were performed in biological duplicate. All adapters and primers used in the ChIP-exo are listed in Table S7 in Data Set S1.

ChIP-exo data analysis was performed as previously described (10). Briefly, sequencing reads were mapped to reference genome assembly R64-2-1 of S. cerevisiae S288C with Bowtie2 (53), and the generated SAM files were converted to sorted BAM files via the removal of low-quality reads. The BAM files were trimmed 70 bases from the 3′ end using trimBam (http://genome.sph.umich.edu/wiki/BamUtil:_trimBam) to increase the resolution. The Integrative Genomics Viewer (IGV) browser (54) was used to visualize the alignment of reads to the genome. The program GEM (20) was used to identify peaks and compare biological duplicates. The noise level was calculated from averaged noise throughout each replicate. Binding events with log_2_(*S*/*N*) ratios of >1 are considered to be reliable, where earlier studies on Chip-exo data have used a log_2_(*S*/*N*) of >1.5 (29), although with the preprocessing of data, some positive events can be lost (Fig. S3). Identification of target genes was done with the *closest* function of BEDTools (55). Gene targets with a distance of more than 1,200 bp from the binding event center were sorted out. The MEME algorithm (56) was used to identify the consensus motifs for all TFs under all conditions. Forty-base-pair sequences were used as input, and the output motifs should have a length between 6 and 15 bp.

To generate the target gene-based ChIP-exo data tables and heat map of binding profiles, we used MatLab (57). The heat map binding profiles were created by extracting the counts of reads 300 bp up- and downstream of the identified binding site. The counts were log_10_ transformed, and the data were then transformed into a heat map profile. All heat map profiles were aligned to center binding.

### Accession number(s).

The ChIP-exo data have been deposited in the Gene Expression Omnibus database under accession number GSE88941.

### Data availability.

Data are also available for viewing at the UCSC Genome Browser.

10.1128/mSystems.00215-17.9 FIG S9  Effect of *OAF1* deletion on the content of fatty acids. The increased contents of short-chain fatty acids (C_12_ and C_14_) and decreased contents of long-chain and very-long-chain fatty acids (C_18_ to C_26_) indicate the downregulation of *ELO1* in the *OAF1* deletion strain. The *oaf1*Δ strain was constructed from strain CEN.PK113-11C using *URA3* as a selection marker. The strains were cultured in minimal medium containing 20 g liter^−1^ glucose and 100 mg liter^−1^ histidine for 72 h. Then, total fatty acids were extracted and analyzed as previously described (58). Data represent means ± standard deviations (SD) for biological duplicates. FAME, fatty acid methyl ester. Download FIG S9, TIF file, 0.5 MB. Copyright © 2018 Bergenholm et al. 2018 Bergenholm et al. This content is distributed under the terms of the Creative Commons Attribution 4.0 International license.
