# Supplementary figures and images for: Reconstruction of a Global Transcriptional Regulatory Network for Control of Lipid Metabolism in Yeast by Using Chromatin Immunoprecipitation with Lambda Exonuclease Digestion
Source: mSystems. 2018 Jul 31;3(4):e00215-17. doi: 10.1128/mSystems.00215-17 (PMC6068829; doi:10.1128/mSystems.00215-17)

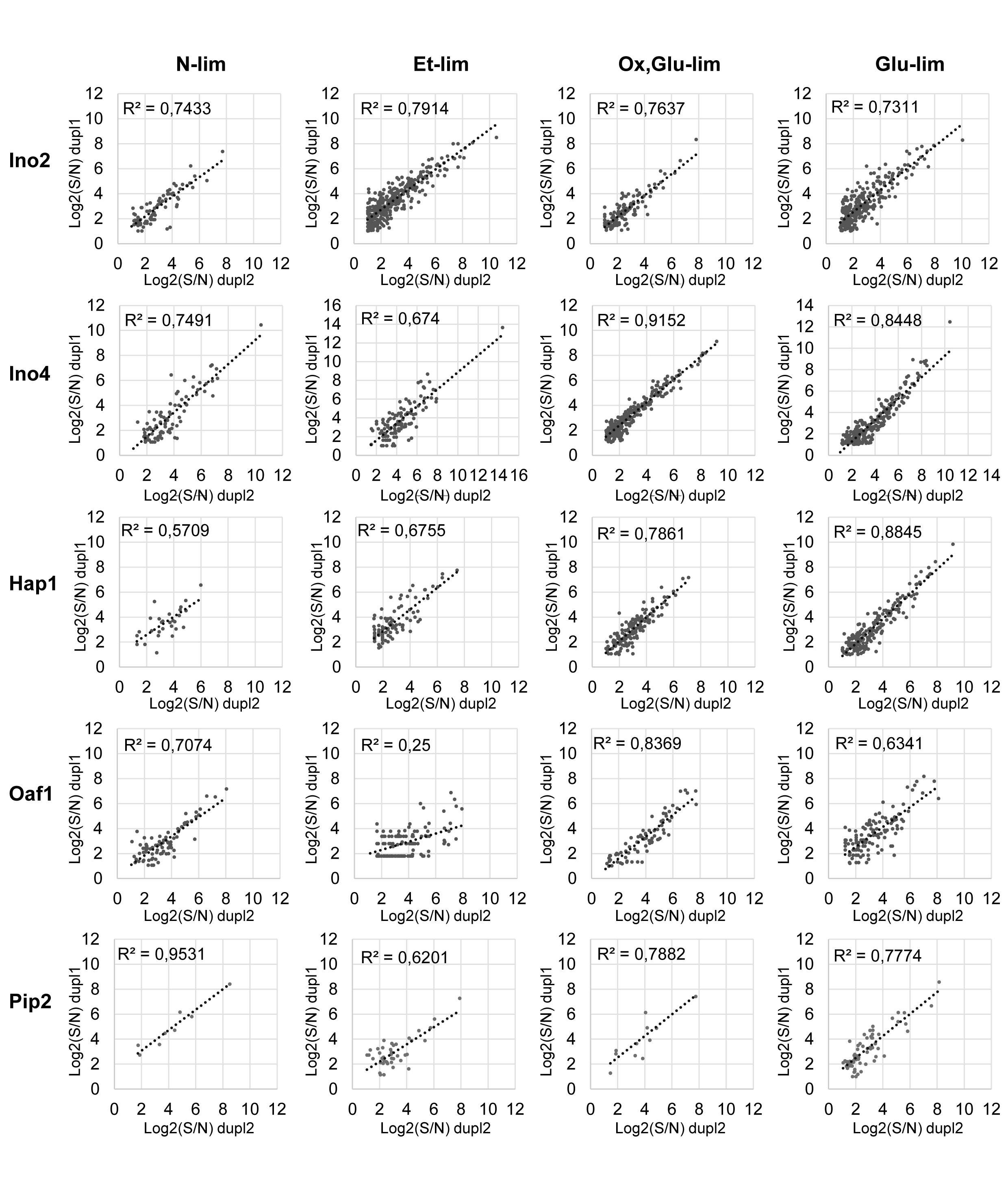

Supplement: FIG S1 [file sys004182252sf1.tif]

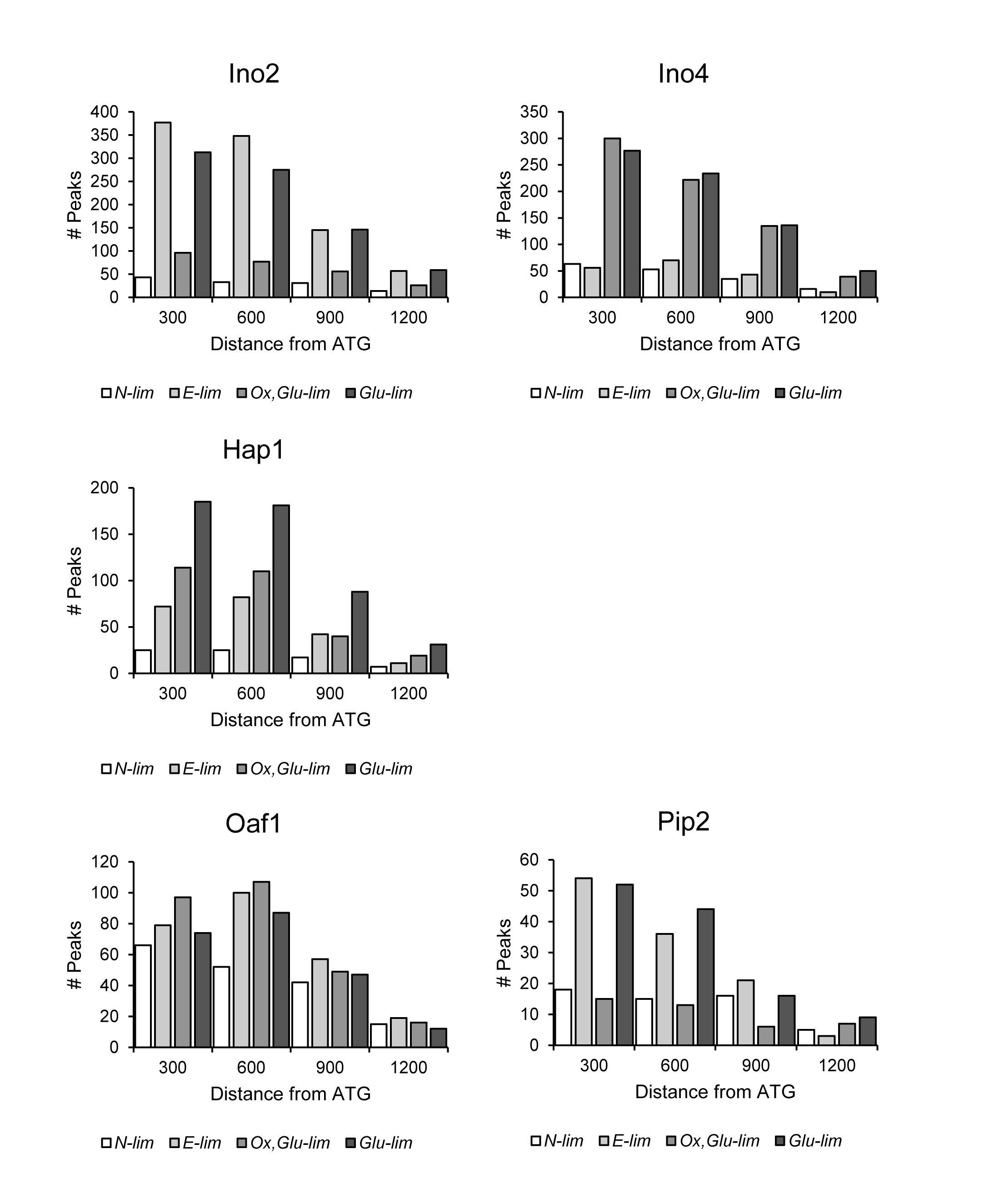

Supplement: FIG S2 [file sys004182252sf2.tif]

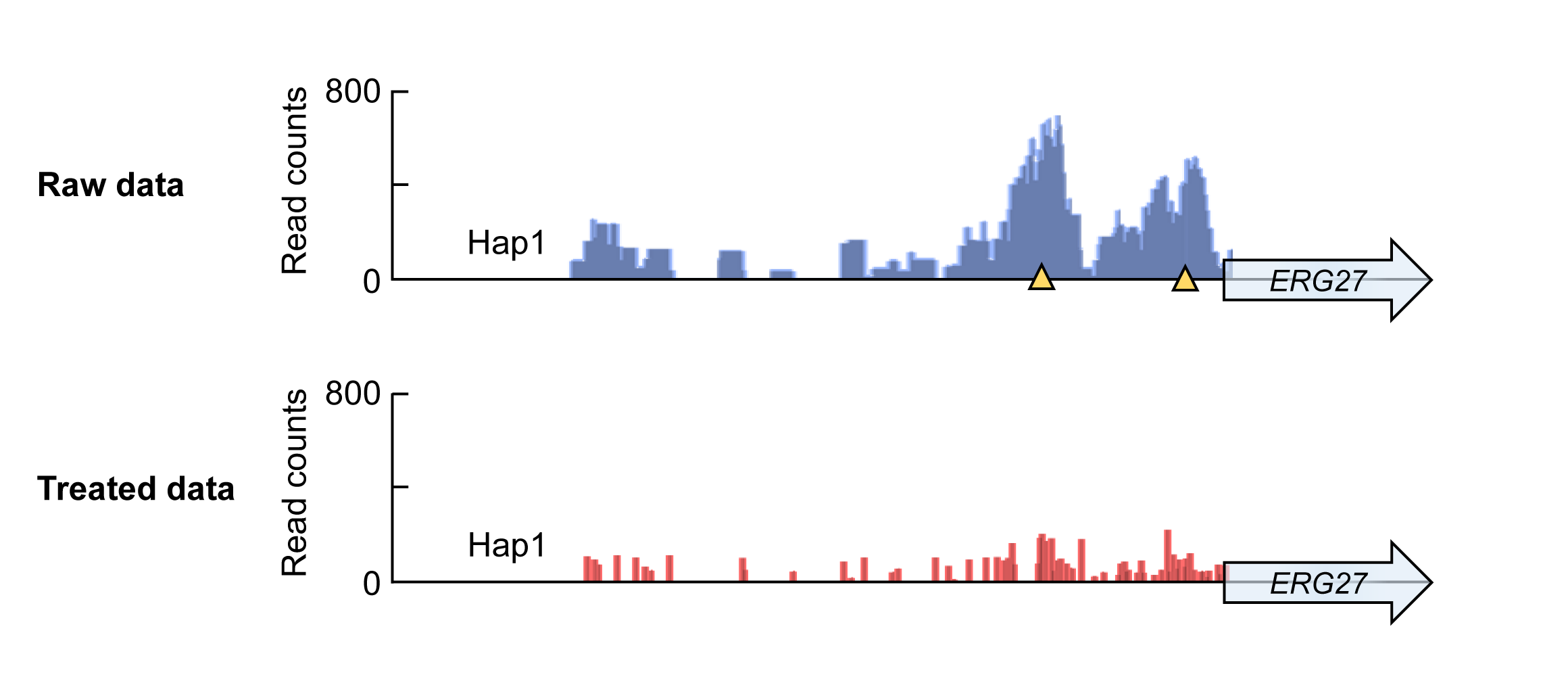

Supplement: FIG S3 [file sys004182252sf3.tif]

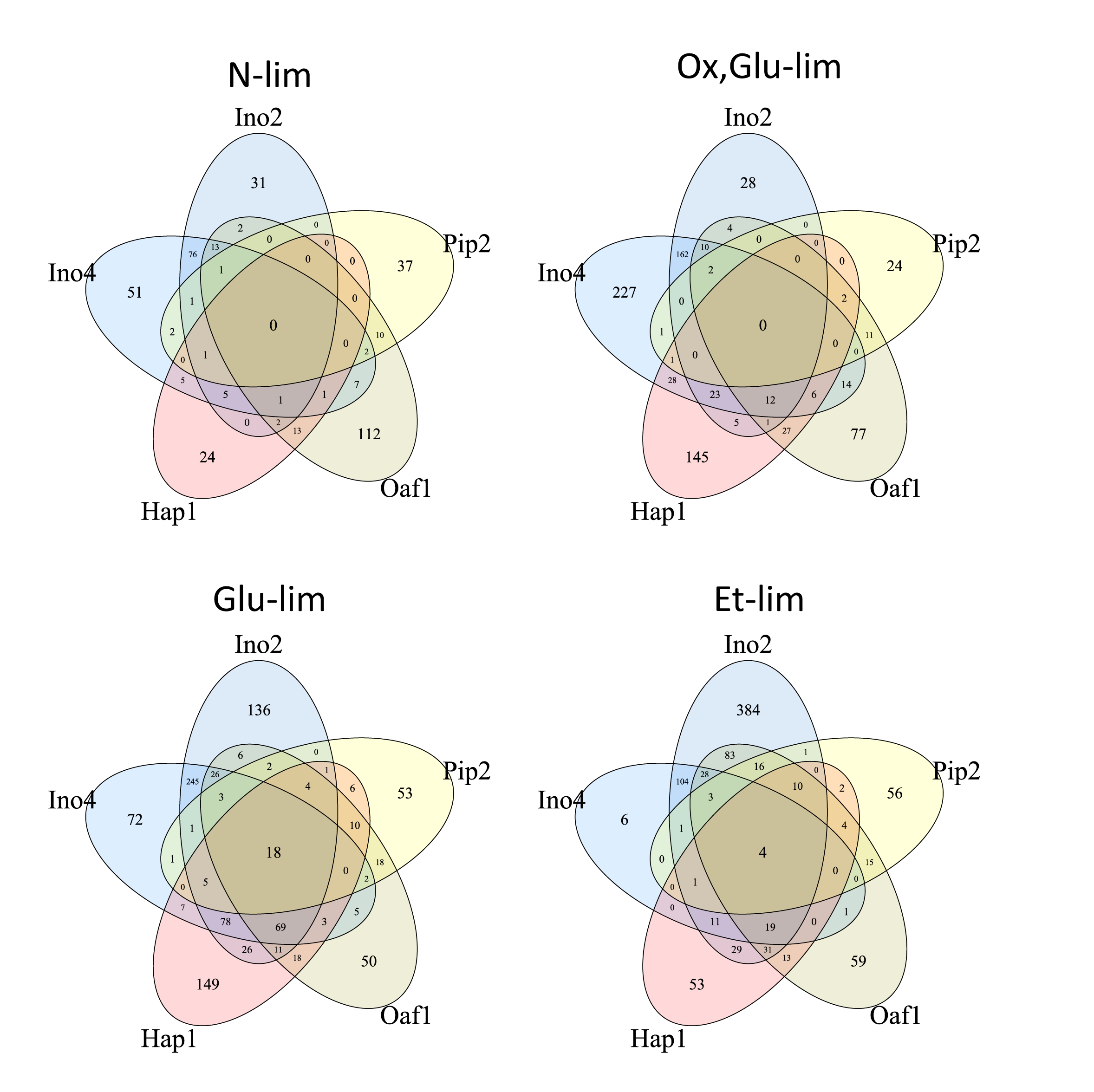

Supplement: FIG S4 [file sys004182252sf4.tif]

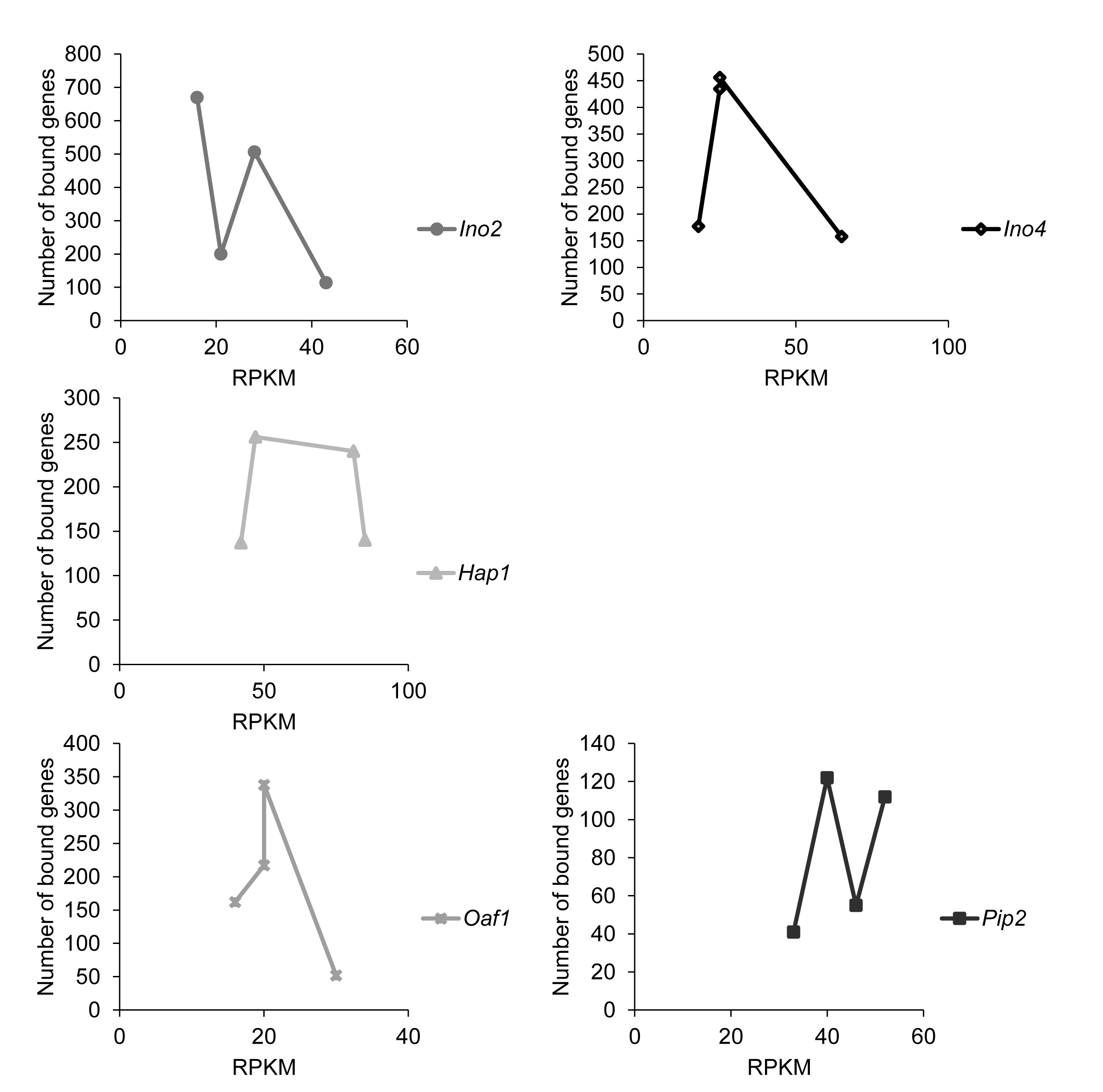

Supplement: FIG S5 [file sys004182252sf5.tif]

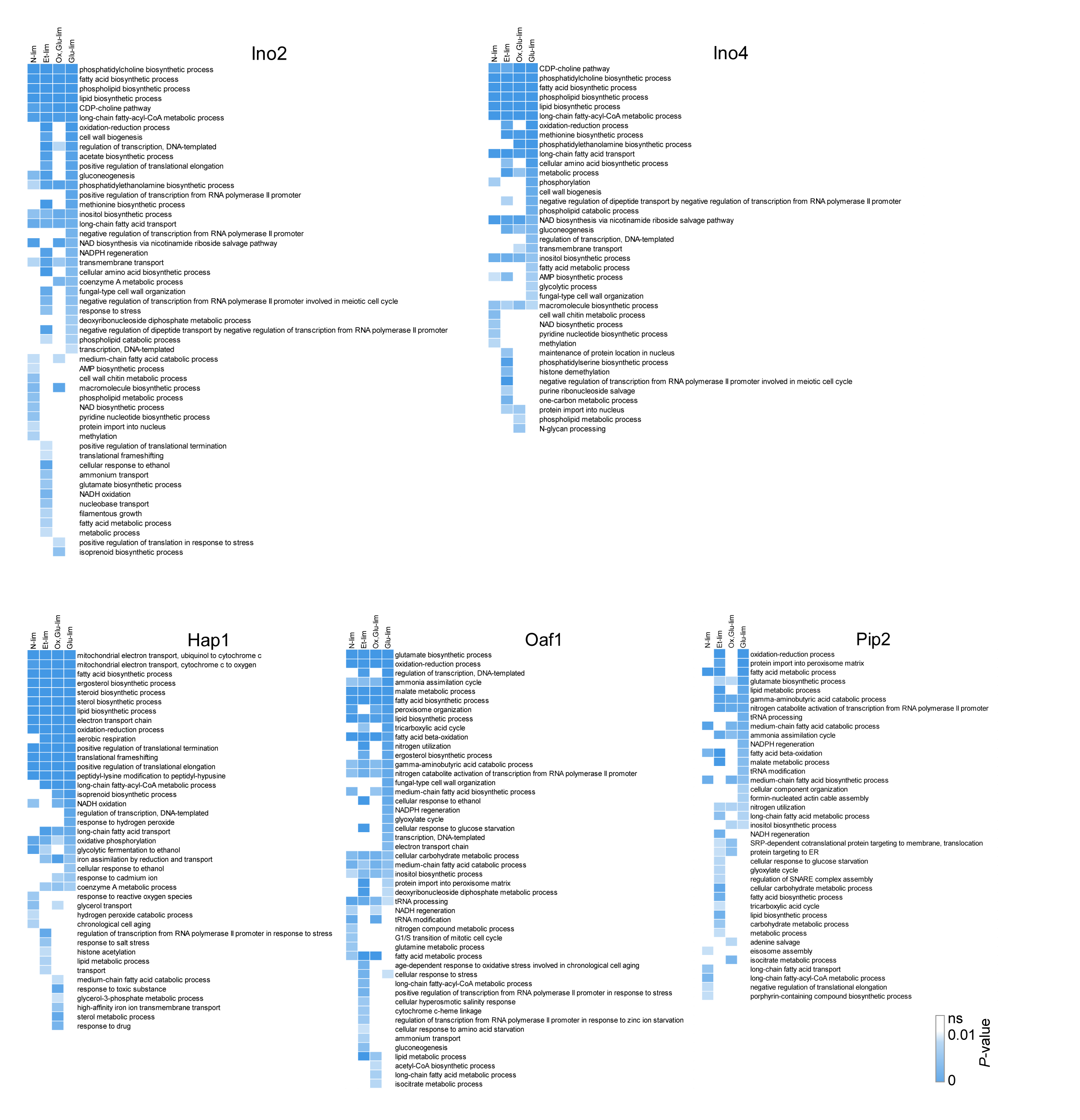

Supplement: FIG S6 [file sys004182252sf6.tif]

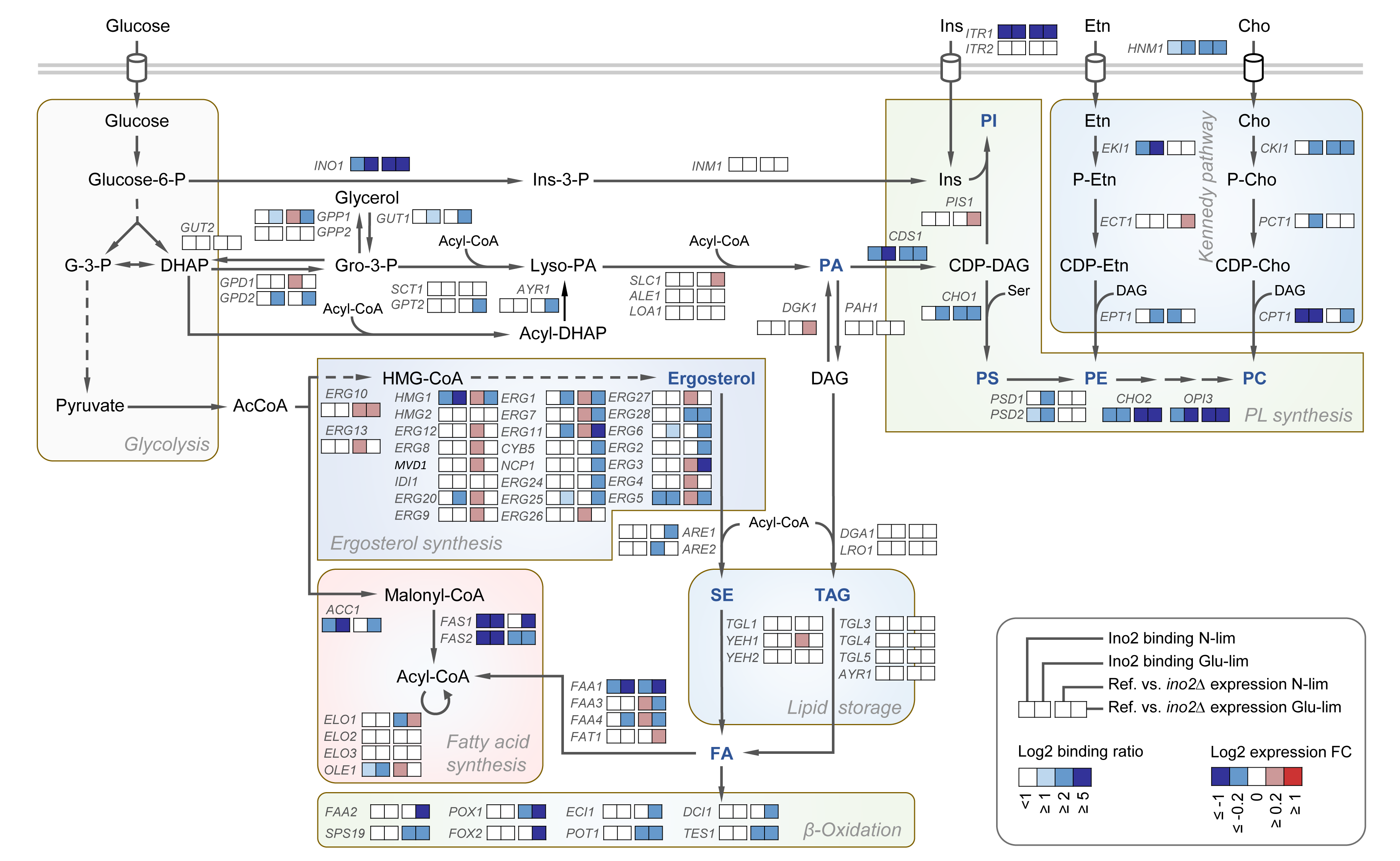

Supplement: FIG S7 [file sys004182252sf7.tif]

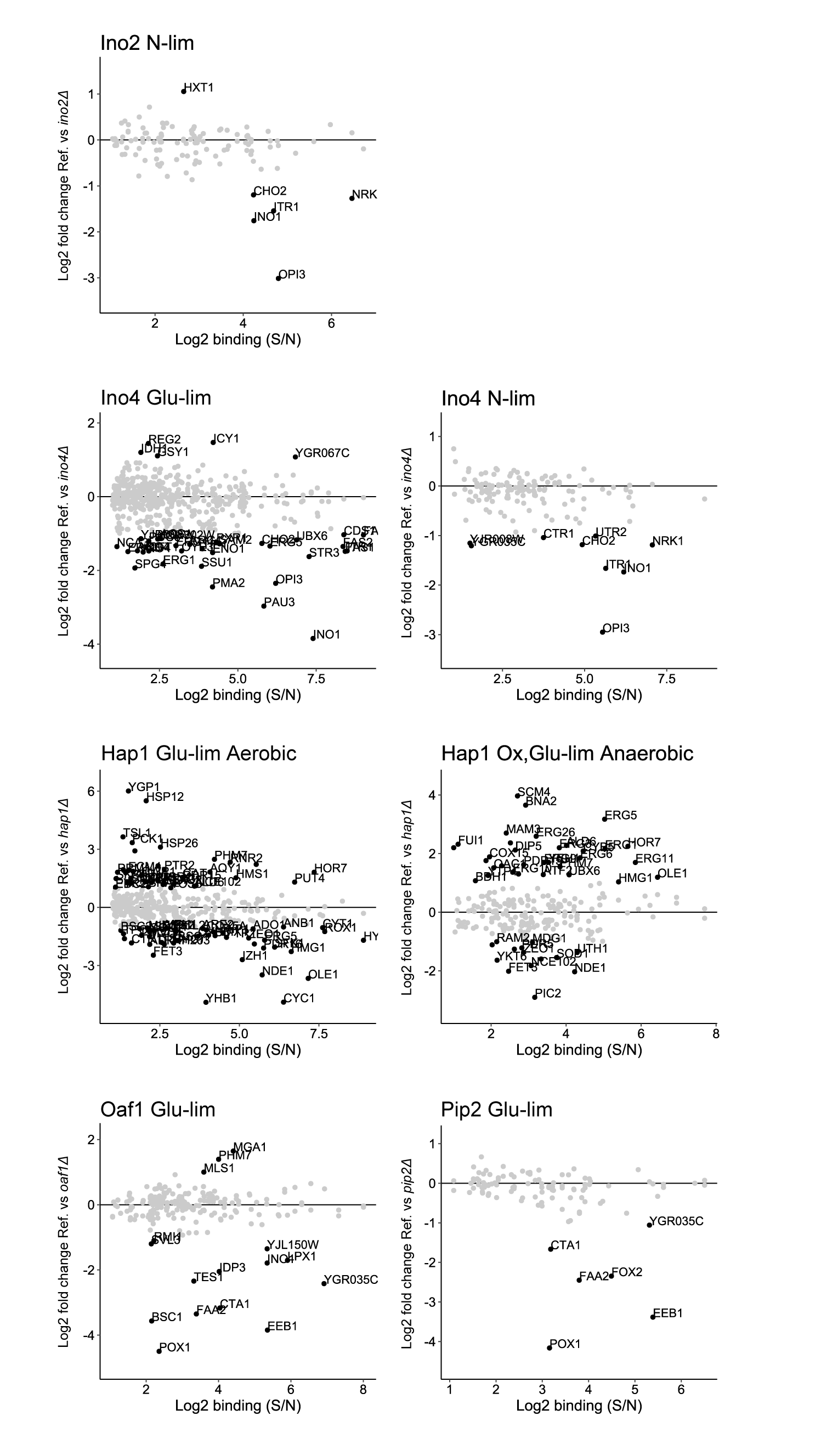

Supplement: FIG S8 [file sys004182252sf8.tif]

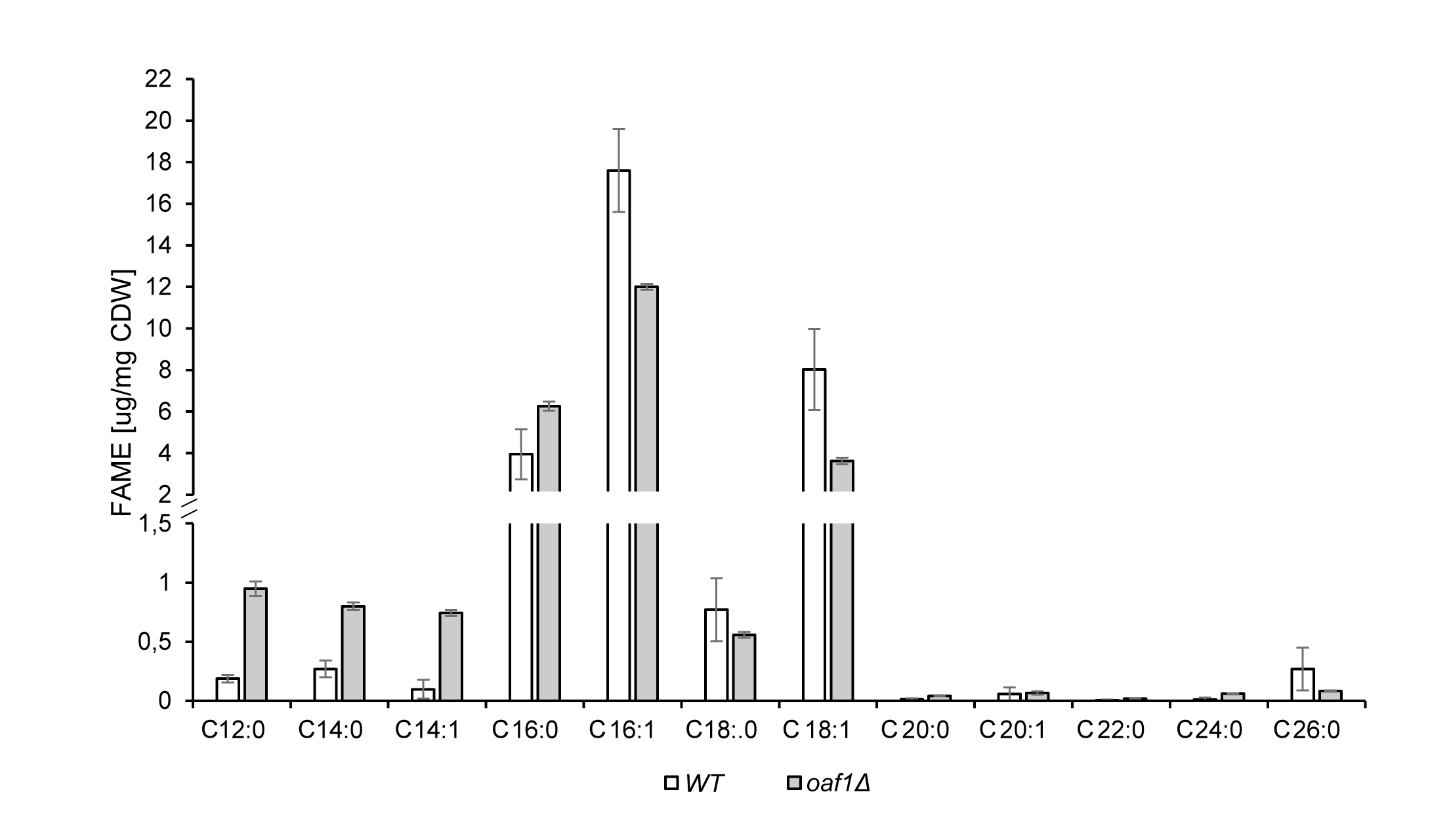

Supplement: FIG S9 [file sys004182252st9.tif]
